# Biomechanical and adhesion comparison of linea alba prophylactic reinforcement with coated and uncoated three-dimensional T-shaped mesh in rabbits^1^

**DOI:** 10.1590/s0102-865020200100000001

**Published:** 2020-11-13

**Authors:** Bruno De Lucia Hernani, Paulo Henrique Fogaça de Barros, Luciano Tastaldi, Luiza Nascimento Ladeira, Sergio Roll, Fabio Gonçalves Ferreira, Diego Paim Carvalho Garcia

**Affiliations:** I Fellow Master degree, Postgraduate Program in Surgery Research, Santa Casa de São Paulo, School of Medical Sciences, and Hospital Alemão Oswaldo Cruz (HAOC), Sao Paulo-SP, Brazil. Acquisition of data, technical procedures, statistical analysis, manuscript preparation and writing.; II MSc, HAOC, Sao Paulo-SP, Brazil. Technical procedures, manuscript preparation.; III MD, University of Texas Medical Branch, Galveston TX, USA. Scientific and intellectual contributions to the study, critical revision.; IV MD, Resident, General Surgery, Hospital Felício Rocho, Belo Horizonte-MG, Brazil. Technical procedures.; V PhD, Associate Professor, Department of Surgery, Irmandade da Santa Casa de Misericórdia de São Paulo, and HAOC, Sao Paulo-SP, Brazil. Scientific, intellectual, conception and design the study.; VI PhD, Associate Professor, Santa Casa de São Paulo, School of Medical Sciences, Sao Paulo-SP, Brazil. Analysis and interpretation of data, critical revision, final approval.; VII PhD, Associate Professor, Department of Surgery, Instituto de Ensino e Pesquisa da Santa Casa, Belo Horizonte-MG, Brazil. Conception and design the study, technical procedures, critical revision, final approval.

**Keywords:** Incisional Hernia, Laparotomy, Surgical Mesh, Abdominal Wall, Tissue Adhesions, Polypropylenes, Rabbits

## Abstract

**Purpose:**

Incisional hernia (IH) is a frequent complication of median laparotomy. The use of prophylactic mesh to reduce IH incidence has gained increasing attention. We hypothesized that in an animal model, linea alba prophylactic reinforcement with a three-dimensional T-shaped polypropylene mesh results in greater abdominal wall resistance.

**Methods:**

Study was performed in 27 rabbits. After abdominal midline incision, animals were divided into three groups according to the laparotomy closure method used: (1)3D T-shaped coated mesh; (2)3D T-shaped uncoated mesh; and (3) closure without mesh. After 4 months, each animal’s abdominal wall was resected and tensiometric tests were applied. Results included IH occurrence, adhesions to the mesh, and wound complications.

**Results:**

There was no significant difference between the groups in maximum tensile strength (p=0.250) or abdominal wall elongation under maximum stress (p=0.839). One rabbit from the control group developed IH (p=1.00). Small intestine and colon adhesions occurred only in the uncoated mesh group (p<0.001) and the degree of adhesions was higher in this group compared to the coated mesh group (p<0.05).

**Conclusion:**

Use of the current 3D T-shaped prophylactic mesh model did not result in a significant difference in tensiometric measurements when compared with simple abdominal wall closure in rabbits.

## Introduction

Midline laparotomy is the most commonly incision to access peritoneal cavity. More than 2 million of these procedures are performed annually in the United States^1^. Incisional hernia (IH) is the main potential complication of this type of surgery, with some challenges to surgeons and patients^1^ ^,^ ^2^. Since the publication of the STITCH trial, the recommended technique for abdominal wall closure is the so-called “small-bites suture”, a continuous suture using either nonabsorbable or slow-absorption monofilament thread with small distances between the stitches and the fascial margin^3^. Even with the appropriate technique, 7.6%–15% will develop IH after laparotomies^4^ ^–^ ^10^. The incidence can reach 45% in high-risk patients who present for example obesity, abdominal aortic aneurysms, or smoking^9^ ^–^ ^14^.

The use of prophylactic mesh in the prevention of IH is not new. Since 1996, several studies describing its use have been published^15^. Recent studies have been published demonstrating benefit of prophylactic meshes in patients with a body mass index >27kg/m^2^ or with abdominal aortic aneurysms undergoing laparotomy^16^. Also, meta-analyses have shown a three-fold increased risk of developing IH in obese patients who have not undergone mesh reinforcement at laparotomy^4^ ^,^ ^7^ ^,^ ^17^.

From a financial perspective, approximately 350.000 incisional herniorrhaphy procedures are performed annually in the United States, with expenses ranging from US $3.800 to US $16.000 per patient^1^. Poulose *et al*.^1^, after analysis of several hospital and financial databases, estimated that every 1% reduction in the incidence of IH would save $32 million annually. Thus, efforts are being made to study and prevent the incidence and recurrence of IH in several patient populations using different mesh types.

The primary objective of the present study was to evaluate the resistance of the linea alba after being reinforced with a 3D T-shaped mesh compared with that sutured without a mesh in rabbits. Secondary objectives were to evaluate and compare visceral adhesions to meshes with and without bovine collagen coating and assess the incidence of wound complications and incisional hernia formation.

## Methods

This study was performed according to the recommendations of the International Convention for the Protection of Animals and the Brazilian Code of Animal Experimentation and approved by the Ethics Commission on the Use of Animals of the Universidade Federal de Minas Gerais (UFMG) under protocol no. 84/2017.

Male New Zealand rabbits aged 3 months and weighing >2 kg were used. The sample size was calculated based on pilot studies, expecting a 50% difference between the groups, using the formula n= 1 + [2C*(s/d)2]^19^. Considering 20% loss margin, the sample size was determined to be 27—three groups of nine animals.

All rabbits were tagged and kept in the *vivarium* of the Faculty of Medicine of UFMG, one per cage. They received rabbit feed daily and filtered water *ad libitum*.

The animals were divided into three groups:

- Group 1: 3D T-shaped uncoated mesh (III), with nine animals
- Group 2: 3D T-shaped coated mesh (IV), with nine animals
- Group 3: Suture only, with nine animals

**Figure 1:**
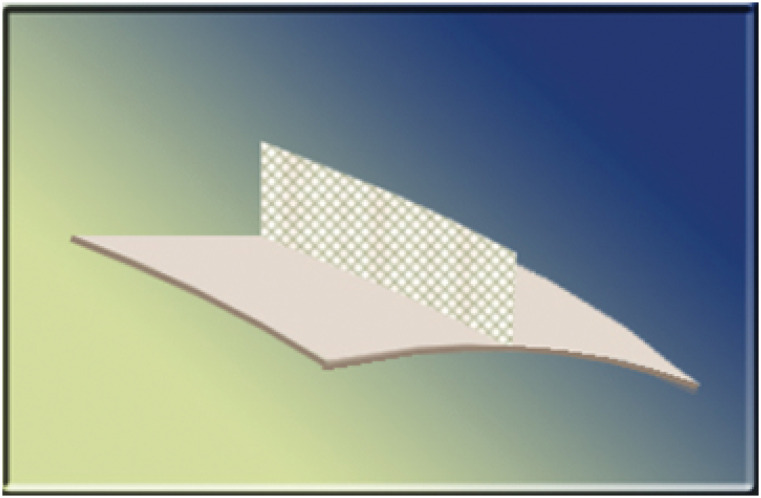
3D mesh in inverted “T” form.

The mesh was developed in collaboration with the authors and was composed of macroporous medium-weight polypropylene. The “T” shape was obtained with a mesh fragment being folded and joined in its upper portion using a laser beam (Fig. 1).

The coated mesh group received a layer of bovine collagen throughout their surface using a dip-coating immersion technique.

### Surgical procedures

Anesthesia was performed for all animals with a gluteal intramuscular injection of 5% ketamine hydrochloride (Ketamin-S® (+), Cristália, Itapira, Brazil) at a dose of 35 mg/kg (0.7 mL/kg) in addition to 2% xylazine hydrochloride (Rompun®, Bayer, São Paulo, Brazil) at a dose of 6 mg/kg (0.3 mL/kg). To detect complications, the heart and respiratory rates were observed throughout the anesthesia period, as were voluntary movements of the rabbit.

The operations were performed in the operating room of the FM-UFMG Central Vivarium in a sterile environment.

**Figure 2:**
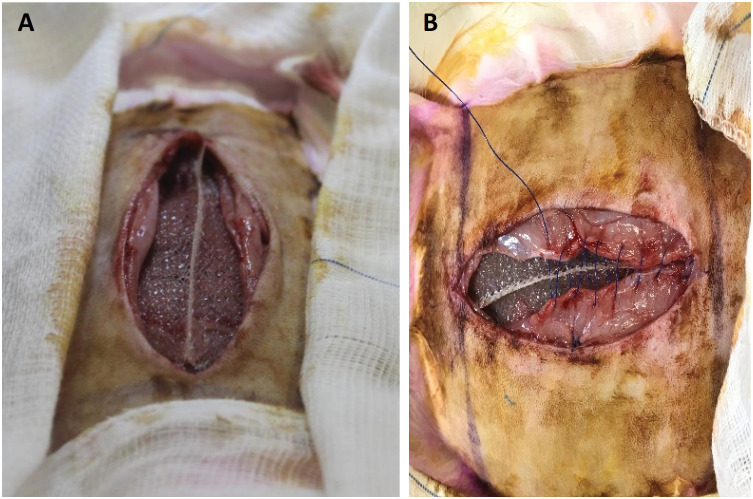
**A.** Mesh in place; **B.** Suture performed in a continuous manner involving the edges and the mesh.

After epilation of the abdominal surface, the surface was disinfected, and surgical fields were placed. Midline laparotomies 10 cm long were performed starting 2 cm below the xiphoid process in the caudal direction. The abdominal walls of animals in group 3 were closed with 3-0 continuous polypropylene sutures (Prolene®, Ethicon, Cincinnati, OH). In groups 1 and 2, the 10 × 3-cm 3D T-shaped mesh of macroporous medium-weight polypropylene, which was uncoated and coated, respectively, was placed between the two edges of the median incision. Six simple stitches were made between each piece of mesh and the abdominal wall, at the four corners of the mesh and at the midpoints between them, to increase the contact area with the parietal peritoneum. The mesh was then sutured together with the incision edges in a continuous manner using 3-0 Prolene®, with each stitch passing through one edge of the wall, then through the mesh in its vertical part and through the other edge, completing the procedure as shown in Figure 2.

After 4 months, the animals were euthanized by carbon dioxide inhalation in a closed chamber after intramuscular injection of 2 mL xylazine (10 mg/kg). A U-shaped laparotomy was performed, releasing a quadrangular portion of the abdominal wall, to observe the occurrence of intra-abdominal adhesions. The following aspects were evaluated according to the adhesion score described by the Surgical Membrane Study Group^20^ (Table 1): intra-abdominal organs with adhesions, presence of vascularization, level of resistance, and percentage of the mesh surface area covered by adhesions.

**Table 1:** Surgical membrane study group adhesion score.

CHARACTERISTICS OF ADHESIONS	SCORE
**Extension of involvement (%)**	
None	0
<25%	1
<50%	2
<75%	3
<100%	4
**Type of adhesion**	
None	0
Filmy, transparent, avascular	1
Opaque, translucent, avascular	2
Opaque, with capillaries present	3
Opaque, with large-caliber vessels present	4
**Tenacity**	
None	0
Adhesions falls apart	1
Lysis of adhesions with traction	2
Lysis of adhesions requiring sharp dissection	3
POSSIBLE TOTAL	11

The abdominal wall was finally removed and prepared for a tensiometric study using Kratos industrial equipment (Cotia, Brazil), model KE200MP, with a 50-kgf load cell.

Initially, all variables were descriptively analyzed. Quantitative variables were presented as minimum and maximum values, and the means, standard deviations, median, and quartiles were calculated. For qualitative variables, absolute and relative frequencies were calculated. In the comparison of the three groups, an analysis of variance by factor was performed; when the assumption of normality of the data was rejected, a non-parametric Kruskal–Wallis test was used, with multiple comparisons performed using Dunn’s test. To verify the homogeneity between the proportions, Fisher’s exact test was used. All statistical analyses were performed using SPSS version 17.0 for Windows. The significance level adopted for the tests was 5%.

## Results

Twenty-six animals survived the experiment and were euthanized. One animal from group 2 died from an infection in the paw, which was unrelated to the procedure.

There was no difference in animal weight and wound complications. One group 3 rabbit developed IH, but this was not statistically significant (p = 1.000).

There was no significant difference between the groups in relation to maximum tensile strength (p = 0.250) or elongation of the abdominal wall under maximum stress (p = 0.839), as shown in Table 2.

**Table 2:** Tensiometric measurements among the groups.

	Group 1	Group 2	Group 3	p
	Mean ± SD []	Mean ± SD []	Mean ± SD []	
Maximum tensile strength (N)	19.92 ± 6.61	21.79 ± 9.66	27.01 ± 10.51	0.250
	(11.28–33.53)	(6.20–37.04)	(11.05–40.93)	
Elongation at the maximum tension (mm)	13.22 ± 3.45	13.00 ± 2.30	13.80 ± 2.70	0.839
	(8.33–18.67)	(9.40–16.45)	(8.01–18.18)	

The degree of visceral adhesions, as measured by the Surgical Membrane Study Group adhesion score, was higher in the group with the uncoated mesh than in the coated mesh (p < 0.05) and control (p < 0.05) groups, as shown in Table 3.

**Table 3:** Adhesion score - Surgical membrane study group (0 to 11).

	Mean	Minimum	Maximum
Group 1	8.44	7.00	10.00
Group 2	5.25	4.00	7.00
Group 3	2.56	0.00	6.00

**Figure 3:**
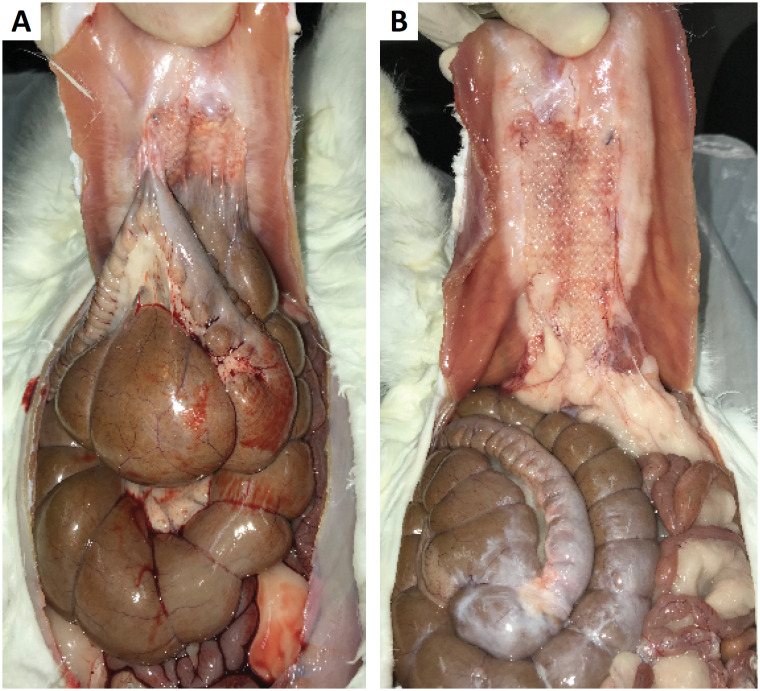
**A.** An example from group 1; **B.** An example from group 2.

Small intestine and colon adhesions occurred only in group 1 (p < 0.001). In addition, the estimated area of adhesion to the mesh (Fig. 3) was significantly lower in the coated mesh group than in the uncoated mesh group (p < 0.05).

Adhesions to the abdominal wall and mesh were observed in all cases with mesh reinforcement but only in 55% of animal in the control group (p = 0.02).

## Discussion

Our study showed that prophylactic linea alba reinforcement using the presented format of 3D T-shaped polypropylene mesh failed to improve resistance in tensiometric tests when compared with simple closure. None of the animals experienced wound complications. Only one instance of IH formation was found in the control group. The adhesions occurred as expected, and the bovine collagen barrier showed to be effective in preventing visceral adhesions.

The prophylactic use of meshes in laparotomy closure remains debatable despite available recommendation (grade of recommendation – 1B) for placement of prophylactic mesh during elective laparotomy in high-risk patients for IH formation^15^ ^,^ ^16^. However, this practice has failed to obtain widespread adoptance, probably due to a lack of technique standardization and concerns for mesh-related complications, including chronic pain, prosthesis infection, seroma formation, in addition of increased cost and operative time. Moreover, a survey study by Bloemen *et al*.^21^ revealed that only 35% of Dutch surgeons close the abdominal fascia using the recommended 4:1 length suture and 68% didn’t change their closure technique since their training, showing that surgeons have some aversion to change their practices.

In a systematic review, Nachiappan *et al*.^22^ concluded that prophylactic placement of a mesh during laparotomy closure in high-risk patients was beneficial, with a significantly reduced incidence of IH and no significant differences in seroma formation and rates of wound infection. However, there was a large variation in surgical procedures because meshes of different materials, absorption capacities, dimensions, and pore sizes were used, as well as various placement and fixation techniques (intraperitoneal, preperitoneal, inlay, and onlay).

Supra-aponeurotic meshes are easy to place but can result in a higher number of wound complications, in addition to presenting a mechanical disadvantage compared with intraperitoneal, preperitoneal, or retromuscular meshes. Retromuscular and preperitoneal meshes require dissection of the intact abdominal wall, in addition to generally increasing operative time^2^ ^,^ ^6^ ^,^ ^7^ ^,^ ^10^.

Since Bellón *et al*.^2^ showed a simple method to reinforce the abdominal wall with a new concept of T-shaped prothesis in rabbits without dissection and no longer operative time, no similar study has been published in the field of increasing abdominal wall resistance. The rationale for vertical placement is that the foreign body reaction caused by the mesh at the edges of the aponeurosis produces more collagen fibers, increasing linea alba resistance.

This T-shaped mesh was easy to place and handle, favoring the reproducibility of the technique. The bovine collagen coating was effective to avoid visceral adhesions. During the traction tests, we observed that the vertical part of the mesh was the site of rupture. Joining the two fragments using a laser beam appeared to have altered the biomechanical properties of polypropylene and did not effectively increase the resistance of the abdominal wall.

We believe that the use of the “T” format would be easy for any surgeon to implant, with no increase in the duration of surgery, no dissection required for placement and the size can be adapted to each patient. Currently, we are working on adjusting the materials and developing a new technique to fabricate the T-shaped mesh to perform further studies.

## Conclusions

The reinforcement of linea alba with this innovative form of polypropylene mesh did not result in a significant difference in abdominal wall resistance when compared with simple closure. The use of this type of mesh coating resulted in minimal adhesions to the mesh in an animal model.
